# Chemotherapy-Induced Peripheral Neuropathy (CIPN): A Narrative Review and Proposed Theoretical Model

**DOI:** 10.3390/cancers16142571

**Published:** 2024-07-18

**Authors:** Kimberley T. Lee, Hailey W. Bulls, Aasha I. Hoogland, Brian W. James, Claudia B. Colon-Echevarria, Heather S. L. Jim

**Affiliations:** 1 Department of Breast Oncology, H. Lee Moffitt Cancer Center and Research Institute, Tampa, FL 33612, USA; 2 Section of Palliative Care and Medical Ethics, Division of General Internal Medicine, Department of Medicine, University of Pittsburgh, Pittsburgh, PA 15213, USA; 3 Department of Health Outcomes and Behavior, H. Lee Moffitt Cancer Center and Research Institute, Tampa, FL 33612, USA; 4 Morsani College of Medicine, University of South Florida, Tampa, FL 33602, USA; 5 Department of Epidemiology, H. Lee Moffitt Cancer Center and Research Institute, Tampa, FL 33612, USA

**Keywords:** chemotherapy-induced peripheral neuropathy, cancer survivorship, theoretical model

## Abstract

**Simple Summary:**

This article outlines a framework for the development and maintenance of chemotherapy-induced peripheral neuropathy using the 3P model, which focuses on predisposing, precipitating, and perpetuating factors. Predisposing factors increase the risk of CIPN onset; chemotherapy triggers the development of CIPN; and perpetuating factors maintain CIPN and limit recovery, such that CIPN becomes a chronic condition. In our proposed model, we have identified demographic, genetic, and clinical factors, such as comorbidities, as predisposing factors. Chemotherapy type, dose, and schedule are precipitating factors. Perpetuating factors include depression and physical activity. The proposed model can be used to identify treatment targets for the management of chemotherapy-induced peripheral neuropathy.

**Abstract:**

Background: Chemotherapy-induced peripheral neuropathy (CIPN) is a common and debilitating symptom experienced by cancer survivors. Despite the burden of CIPN-related symptoms, interventions remain limited. Objectives: This narrative review seeks to propose a framework for CIPN predisposing, precipitating, and perpetuating factors (3Ps), which will provide a foundation for future research and clinical interventions aimed at mitigating CIPN-related symptoms and morbidity. Methods: A comprehensive literature search was performed using PubMed, guided by keywords related to “chemotherapy-induced peripheral neuropathy.” Studies were limited to those with full text available in English. Results: Predisposing factors outlined in this framework, such as older age and comorbid conditions, can be used to identify patients who have a higher risk of developing CIPN. The major precipitating factor of CIPN is the delivery of chemotherapy to peripheral nerves, which may be mitigated via cryotherapy or compression therapy during chemotherapy. Perpetuating factors can offer insight into psychological, cognitive, and behavioral modifications that could be treatment targets for CIPN management. Conclusion: The proposed 3P model can guide the development of effective interventions for CIPN by suggesting modifiable psychological and behavioral treatment targets that may mitigate the impact of CIPN for cancer patients.

## 1. Introduction

Chemotherapy-induced peripheral neuropathy (CIPN) is a common physical side effect of cancer treatment that is often debilitating. CIPN affects up to 90% of patients treated with taxanes (e.g., paclitaxel and docetaxel), platinum agents (e.g., carboplatin and oxaliplatin), vinca alkaloids (e.g., vincristine), and proteosome inhibitors (e.g., bortezomib) [1,2]. These chemotherapeutic agents are commonly used in the treatment of breast cancer, prostate cancer, colorectal cancer, gynecological malignancies, testicular cancer, and blood cancers, such as multiple myeloma. Thus, CIPN is extremely common among cancer patients.

CIPN is characterized by a variety of sensory and motor symptoms, including burning/shooting pain, numbness, tingling, a reduced sense of touch, reduced proprioception, weakness, poor balance, and deficits in fine motor skills [3]. CIPN is typically managed via chemotherapy dose reductions, treatment interruption, or discontinuing therapy [4]. As such, chemotherapy dosage reductions due to CIPN can have negative effects on cancer progression and survival outcomes, such as decreasing the efficacy of curative-intent adjuvant treatment for breast cancer or colon cancer [5]. Furthermore, CIPN can have a profound and lasting impact on patients’ quality of life after the discontinuation of the causal chemotherapy agent; for up to 42% of patients, CIPN becomes chronic and unremitting [6]. Across the cancer continuum, CIPN is associated with reduced quality of life and a constellation of negative psychological and behavioral sequelae [7,8,9,10]. Patients often note that CIPN is distressing and can limit everyday activities, such as buttoning shirts, using remote controls, and walking [11].

To our knowledge, there is no comprehensive theoretical model of CIPN. Without such a model, it is difficult to identify patients who are at high risk for CIPN development, fully understand the impact of CIPN symptoms on patients’ lives, effectively assess CIPN and its associated functional limitations, and develop targeted interventions to alleviate the burden of CIPN. A better conceptualization of the contributors to and consequences of CIPN is critical to furthering our research in these areas, as preventative and treatment options for CIPN are limited.

This narrative review seeks to synthesize the existing literature on CIPN, identify gaps in our understanding of the condition, and propose a theoretical framework that integrates predisposing, precipitating, and perpetuating (3P) factors. The 3P model has successfully been used to understand other cancer-related symptoms such as insomnia and fatigue [12,13,14]. This 3P model will provide a foundation for future research and clinical interventions aimed at mitigating CIPN-related symptoms and morbidity.

## 2. Materials and Methods

**Figure 1:**
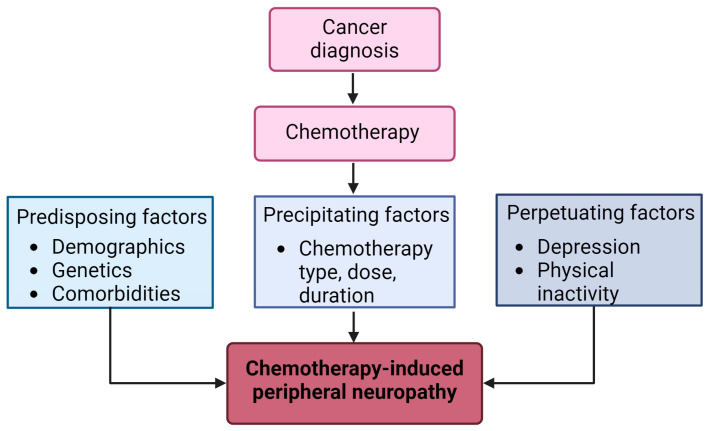
3P model of chemotherapy-included peripheral neuropathy (Created using Biorender).

The development of the 3P model of CIPN began with a review of the literature on cancer-related pain, fatigue, and insomnia. Based on this review, theoretical candidates for CIPN risk were chosen as potential contributors to the development and maintenance of CIPN in the context of the 3P model. This transdisciplinary team of coauthors with expertise in cancer, symptom management, and patient-reported outcomes finalized the model based on the existing literature and expert opinion and created a figure to represent the 3P CIPN model (Figure 1). Within the 3P model, predisposing factors can be broadly defined as any factor that increases the risk of CIPN onset. Conceptually, predisposing factors for CIPN may include sociodemographic, clinical, and biological characteristics. Chemotherapy is then the precipitating factor, or trigger, for CIPN development. Finally, perpetuating factors are those that maintain CIPN and limit recovery, such that CIPN becomes a chronic condition. For example, behaviors like sleep disturbance and physical inactivity may maintain or worsen CIPN symptoms over time. Taken together, these predisposing, precipitating, and perpetuating factors may coalesce in a “perfect storm” that propels CIPN into a years-long, chronic condition.

## 3. Results

### 3.1. Methods of Assessment

Patient-reported outcomes (PROs) are increasingly used to assess symptom burden among oncology patients. This effort has resulted in the development of several self-reported CIPN measures, including the Functional Assessment of Cancer Therapy (FACT) neuropathy subscales and the European Organisation for Research and Treatment of Cancer Quality of Life Questionnaire and Chemotherapy-Induced Peripheral Neuropathy 20 (EORTC-CIPN20) [7,15]. In both of these questionnaires, patients are asked to score their sensory, motor, and autonomic symptoms on an ordinal scale. Validation studies have found these measures to be valid, reliable, and sensitive to changes in CIPN over time, though these studies are often limited to one population at a time (e.g., women with gynecologic cancer) [16,17,18].

However, despite the utility of PROs in research, formal self-report measures are not often used to assess CIPN in clinical practice [19]. In clinical research, CIPN is commonly reported via the National Cancer Institute’s Common Terminology Criteria for Adverse Events (CTCAE v.5) grades for neuropathy: (1) asymptomatic (clinical or diagnostic observations only); (2) moderate symptoms, which limit instrumental activities of daily living such as food preparation or housekeeping; (3) severe symptoms, which limit self-care activities of daily living; (4) life-threatening consequences, which indicate a need for urgent intervention; or (5) death. However, CIPN is not always formally assessed in clinical practice because of time constraints, with physicians often only formally assessing severe patient-reported symptoms [20]. Thus, mild CIPN is less likely to be captured on a physician’s assessment [21]. Additionally, research suggests that provider-based assessments may underestimate the severity of CIPN symptoms when compared to patient reports [22]; Nyrop et al.’s comparison of patient-reported and clinician-assessed CIPN scores demonstrated minimal agreement. For example, of the 65 patients who reported grade 1 CIPN, 31% of clinicians rated their CIPN as grade 0. Similarly, for the 47 patients who reported grade 2 CIPN, 51% of the clinician-rated CIPN scores were rated lower as grade 0 or 1 [22].

Objective testing can be combined with clinician assessment and/or PROs to improve sensitivity and reliability. This may include quantitative sensory testing (e.g., heat, cold, and pressure sensitivity) and intraepidermal nerve fiber density evaluations. For example, the Total Neuropathy Score (TNS) includes pin prick, vibration threshold, and nerve conduction testing, combined with subjective reporting of sensory, motor, and autonomic items [23]. Validation of the TNS indicated that the measure was highly correlated with the NCI-CTCAE grade and was more sensitive than physician assessment in capturing severe CIPN symptoms. However, despite the validity of the TNS, it was also burdensome to conduct. The full TNS requires approximately an hour to complete, and many clinical practices are not equipped to perform nerve conduction assessments [19]. As a result, objective CIPN measurement is likely underused, especially in oncology practices, and more work is needed to develop efficient and clinically useful objective testing procedures. Though objective testing is valuable, it is important to note that subjective assessments are more likely to capture the impact of CIPN on a patient’s functioning and quality of life, and they may therefore be the most clinically relevant measure [24]. Notably, there is no scientific consensus on the most valid approach for CIPN measurement, and approaches vary widely, which limits our ability to directly compare existing studies [19].

### 3.2. Predisposing Factors

Predisposing factors refer to characteristics that make a patient vulnerable to developing CIPN. Here, we provide an overview of this literature, including sociodemographic characteristics, such as age, race/ethnicity, and biological sex; clinical characteristics, such as pre-existing conditions, prior chemotherapy, and obesity; and biological characteristics, such as germline genetic variations.

#### 3.2.1. Age

In general, older patients treated with chemotherapy have higher rates of severe treatment toxicities [25,26], with up to 60% of older adults experiencing a grade 3 or 4 toxicity [26,27,28,29]. Toxicities can result in frequent dose reductions, delays in treatment, and unplanned hospitalizations. In one series of 550 older adults treated with chemotherapy, 28% required dose reductions and 37% required dose delays [28]. Chemotherapy-related toxicities are also associated with high rates of unplanned hospitalizations, with estimates ranging from 27% to 37% [29,30]. However, literature examining the effect of age on CIPN specifically shows mixed results.

We identified eight studies that addressed the relationship between age and CIPN [4,31,32,33,34,35,36,37]. Of these, three demonstrated that older age was associated with an increased incidence or severity of CIPN, and four showed no significant relationship. Interestingly, one study of 425 cancer patients with persistent posttreatment CIPN showed that older age may be a protective factor, with older patients reporting less severe pain in the hands and feet and less interference with routine activities than younger patients [36].

In an effort to understand these discrepancies, our group previously conducted a longitudinal study of 90 women with gynecologic cancer who were followed for treatment-related side effects during treatment and up to a year posttreatment [38]. Patients reported CIPN symptoms via the EORTC-CIPN-20, which was conducted three times during chemotherapy, once during their 6-month posttreatment follow-up, and once at their 12-month follow-up. Medical record reviews were also conducted to abstract clinician-documented CIPN. Piecewise mixed models revealed that older and younger patients reported similar increases in CIPN during the active treatment phase. However, older patients tended to not recover from CIPN after treatment completion, whereas younger patients reported significant improvement in CIPN symptoms posttreatment. No group differences were observed in the presence of provider-recorded sensory neuropathy and pain. Thus, we concluded that older adults may have a higher risk for chronic CIPN, requiring additional education and treatment.

Little is known about the biological underpinnings of the relationship between age and CIPN. Aging is associated with a variety of peripheral nerve changes, including slower nerve conduction velocity and increased sensory fibers with signs of damage or degeneration; this may put older patients at increased risk for developing CIPN [39,40], and the impact may be greater for older patients if there are preexisting limitations in functional status. In addition to biology, important methodological considerations may impact the outcomes of studies focused on aging and CIPN, such as how (objective vs. patient-reported) and when (e.g., during or after treatment) CIPN symptoms are assessed. Finally, psychosocial factors may also help explain findings related to aging and CIPN. For example, older patients may be more accepting of chronic pain and may manage their expectations for pain relief more adaptively than younger patients, which can help to modulate reported pain intensity [36].

#### 3.2.2. Biological Sex

Though sex-related differences have been observed in various types of clinical pain conditions [41,42], few studies support the idea that biological sex influences the onset or maintenance of CIPN. In a pooled analysis of randomized clinical trials for gastrointestinal cancers treated with a platinum agent, men were more likely than women to develop any grade of CIPN, but there were no sex-based differences for more severe grade 3 or higher CIPN [43]. However, two studies of patients with multiple myeloma showed no association between sex and CIPN [44,45]. Given the scarce literature in this area, the effect of biological sex on CIPN trajectories, if any, has yet to be determined.

#### 3.2.3. Race and Ethnicity

Racial and ethnic differences have been identified in a wide range of chronic pain conditions, including post-surgical pain [46], knee osteoarthritis [47,48], and HIV/AIDS pain [49]. There is also growing evidence of racial/ethnic disparities in the development of CIPN. In a study comparing outcomes among colorectal cancer survivors relative to other cancer types, Black patients were more likely to report moderate-to-severe numbness and tingling than their White counterparts, though neuropathic pain was not specifically evaluated [50]. Additionally, in a group of women previously treated with taxane-based chemotherapy for early-stage breast cancer, Black women reported a higher severity of CIPN-related sensory symptoms [51]. Though an analysis of a clinical trial for breast cancer found no statistically significant increase in risk for development of patient-reported CIPN among Black women receiving taxane chemotherapy compared to non-Black women [35], a subsequent analysis of this trial data using genetically determined race demonstrated that Black race was associated with an increased risk of CIPN development [52].

In an effort to understand the mechanisms of increased risk for CIPN, Schneider et al. evaluated germline genetic variants in Black breast cancer survivors treated with taxane-based chemotherapy. Their findings indicated that rare variants in SET binding factor (*SBF*2) were significantly associated with CIPN development [53]. *SBF2* is also associated with Charcot–Marie–Tooth disease, a form of hereditary polyneuropathy. A comparison of genetic or other biological predispositions that may be more prevalent among African-American patients may provide unique insight into CIPN mechanisms; however, the current literature in this area is limited.

#### 3.2.4. Patient Clinical Characteristics and Comorbidities

Notably, some studies suggest that CIPN and an overall burden of comorbidities are positively associated, perhaps indicating that overall worse health is indicative of increased risk for worse CIPN trajectories (and/or that CIPN reciprocally results in worse overall health) [54]. Conceptually, several pre-existing conditions may exacerbate the likelihood of developing CIPN, including prior cycles of neurotoxic chemotherapy, uncontrolled diabetes mellitus, alcohol consumption, and inherited neuropathic conditions (e.g., Charcot–Marie–Tooth disease) [37,55,56,57,58,59]. Additionally, several studies have examined the relationship between obesity and CIPN, with the majority of results showing significant positive correlations [60,61,62]. Because obesity is associated with a worse cancer prognosis overall and subsequently with potentially more neurotoxic therapies, more research is needed to understand the mechanism of this relationship [61].

### 3.3. Precipitating Factors

The precipitating factor for CIPN is chemotherapy itself. CIPN severity and its constellation of related symptoms may depend on characteristics of the treatment regimen and patient-related predisposing factors, such as prior surgery [63,64,65], autoimmune conditions [66], Raynaud’s phenomenon [67], hypoalbuminemia [68], anemia during treatment [60,68], and decreased creatinine clearance [54]. Here, we highlight platinum-based chemotherapy and taxane therapy, two common regimens associated with CIPN, and the treatment characteristics that may impact CIPN.

#### 3.3.1. Chemotherapy Type: Platinum-Based Chemotherapy

Platinum-based chemotherapy includes cisplatin, carboplatin, and oxaliplatin. Cisplatin is commonly used to treat bladder, ovarian, and testicular cancers; carboplatin is primarily used to treat breast, lung, and ovarian cancers; and oxaliplatin is typically used to treat colorectal cancer [69]. The exact mechanism of action by which platinum-based chemotherapies cause CIPN is debated, with leading theories believing it is due to damage to the axons of either small-nerve fibers [70] or large-nerve fibers [71]. However, it is clear that platinum-induced acute CIPN is common. Estimates suggest that acute CIPN occurs in up to 89% of patients [72,73], with symptoms typically peaking 3 days after each individual infusion and worsening cumulatively over the duration of treatment [73,74]. Symptoms are typically severe [69,75], occur primarily in the upper extremities, and are characterized mainly by sensory deficits (e.g., tingling and numbness) [56,76,77]. CIPN resulting from platinum-based chemotherapy may continue to increase in the months after treatment cessation until slow relief occurs naturally over time, a phenomenon termed “coasting” [58,73,74].

#### 3.3.2. Chemotherapy Type: Taxanes

Taxane-based chemotherapy includes paclitaxel and docetaxel, which are commonly used to treat breast, gynecological, prostate, gastric, and head and neck cancers [69]. The mechanism of action by which taxane-based chemotherapies cause CIPN is theorized to be the same as that of platinum-based chemotherapies (i.e., damage to axons of either small-nerve [70] or large-nerve fibers [71]). Taxane-induced CIPN occurs in up to 80% of patients [69] and is reported more frequently among patients treated with paclitaxel than docetaxel [51,63,78,79].

Like platinum-induced neuropathy, taxane-induced neuropathy causes a predominantly sensory-focused CIPN, including numbness, tingling, burning, sensory loss, and, rarely, motor deficits [80,81]. Symptoms typically peak the third day after each individual infusion, but unlike platinum-based CIPN, the severity of CIPN symptoms is similarly intense during each cycle and equally affects the upper and lower extremities [73,74]. Independent of CIPN and potentially related to this pattern, taxane-based chemotherapies may be more likely to result in falls than platinum-based chemotherapy [82]. Coasting (i.e., symptoms continuing to increase after treatment cessation) is not typically seen with taxane-based chemotherapy, with acute CIPN usually improving immediately after chemotherapy cessation [73,74]. However, up to 35% of patients experience chronic CIPN long after the conclusion of treatment [55,75].

#### 3.3.3. Chemotherapy Characteristics: Dose, Intensity, and Duration

Beyond regimen type, several aspects of the chemotherapy treatment course are associated with CIPN symptoms, including dose per cycle or dose intensity, cumulative dose, and duration of infusion. Multiple studies of taxanes have demonstrated higher rates of CIPN with higher doses per cycle when cycle length was kept the same [83]. A narrative review of several studies reported that, on average, increasing the cumulative dose of the chemotherapy agent rises is positively associated with increased incidence and severity of acute CIPN [83]. Cumulative dosage has also been associated with chronic CIPN symptoms persisting months to years past the conclusion of chemotherapy, though the literature is mixed [84]. For example, one study examining colorectal cancer patients 2 to 11 years after the conclusion of treatment found that patients with higher cumulative doses of oxaliplatin reported worse persistent sensory symptoms, including numbness and tingling in their feet and toes, than their counterparts with lower cumulative doses [85]. Generally, chemotherapy delivery over longer periods of time is associated with a lower incidence of CIPN symptoms [86]. For example, taxane-induced CIPN was observed in 13% of patients when chemotherapy was delivered over 3 h, whereas the same dosage delivered over 24 h resulted in a CIPN incidence rate of only 6% [75].

These relationships between chemotherapy agent, dosage, duration, and CIPN are concerning, as they suggest that the most severe CIPN cases are likely to occur among patients who require the longest and most intense treatments. Given the relative lack of efficacious management strategies, severe CIPN is often treated by delaying or discontinuing chemotherapy entirely [75]. Early discontinuation of chemotherapy has been associated with worse cancer outcomes [87,88]. As such, there’s no clear solution on whether it’s better to continue treatment despite severe CIPN or to alter treatment and risk the patient’s survival.

More research is needed to identify which chemotherapy regimens maximize the likelihood of survival while minimizing the impact of CIPN. Given the potentially debilitating effects of CIPN, including chronic pain and decreased mobility, it is important to develop and test assessment tools for real-life monitoring of the development of CIPN during chemotherapy. Early detection of CIPN may result in earlier interventions, which may ultimately limit CIPN-related toxicity, but this is deserving of further study. In addition, effective treatments are needed to manage CIPN and maximize chemotherapy tolerance, which may ultimately improve survival. Finally, a better understanding of the underlying pathophysiology of CIPN is needed to develop less harmful treatments and avoid CIPN entirely.

### 3.4. Perpetuating Factors

Perpetuating factors refer to those that maintain CIPN over time, after the precipitating factor is removed. It is difficult to pinpoint the directionality of relationships between CIPN and perpetuating factors, as well as precisely when those factors may start in the cancer trajectory. Notably, patients may engage in poor health behaviors in an effort to manage acute CIPN symptoms (e.g., sedentary behavior may be used to cope with painful walking and fear of tripping/falling), only for these behaviors to maintain long-term CIPN symptoms. Given the complexity of painful conditions like CIPN, there are likely many perpetuating factors. Here, we describe two of the most commonly documented perpetuating behaviors associated with CIPN: lack of exercise and sleep disturbance.

#### 3.4.1. Lack of Exercise

Exercise is thought to reduce chronic inflammation by downregulating the inflammatory milieu that contributes to CIPN [89]. Chronic exercise increases endogenous antioxidant signaling and has been demonstrated to increase anti-inflammatory signaling, which has the downstream effect of decreasing nociceptive excitability [89,90,91]. Emerging research suggests that engaging in exercise during and after cancer treatment may have a positive impact on symptoms related to CIPN. In a recent systematic review and meta-analysis, exercise was shown to reduce neuropathic pain in cancer survivors with CIPN [92,93,94]. However, a meta-analysis of four exercise intervention studies showed no significant difference in CIPN symptoms between patients in the exercise and control groups [94,95,96,97,98]. The observed differences in the impact of exercise on CIPN severity may be due to the heterogeneity with which exercise is defined in these studies, particularly in terms of exercise type (e.g., endurance training, balance training, or resistance training), intensity, timing (e.g., before, during, or after chemotherapy), and duration [94].

The data regarding the relationship between exercise and CIPN are modest, and this is reflected in the recommendations from the American Society of Clinical Oncology (ASCO) and the European Society for Medical Oncology (ESMO), the two major oncologic professional societies pertaining to clinical care. ASCO makes no recommendation for exercise for the prevention or treatment of CIPN [99], whereas ESMO states that exercise can be offered to reduce the risk of CIPN development and to treat CIPN at the onset of symptoms [100].

#### 3.4.2. Sleep Disturbance

Though the research is limited, cross-sectional studies suggest that sleep disturbance and CIPN may co-occur across the cancer continuum [89]. A cross-sectional study of 706 patients with various cancer diagnoses found that CIPN was associated with worse sleep quality after controlling for demographic and disease-related confounders [10]. Another large cross-sectional study demonstrated a positive relationship between pain sensitivity and the frequency and severity of insomnia [101]. Patterns of sleep disturbance and CIPN symptoms appear to be similar after the conclusion of treatment. Among three studies of posttreatment patients with colorectal cancer [102], breast cancer [103], and ovarian cancer [104], every study suggested that worse sleep disturbance symptoms were associated with higher CIPN severity, which persisted, in some cases, up to 12 years after the conclusion of treatment.

Our understanding of this relationship is limited by the largely cross-sectional nature of the studies described above. Previously, our group used a longitudinal design to examine relationships during and after treatment in gynecologic cancer patients at approximately 6-month intervals (at the start of chemotherapy, immediately after chemotherapy conclusion, and 6- and 12-months after treatment). Results of this analysis found that self-reported sleep quality exerts a directional influence on subsequent self-reported CIPN, such that patients with worse sleep quality reported higher CIPN symptoms, but not vice versa [105]. Additional research is needed to replicate these findings across a broader group of cancer patients. However, taken together with cross-sectional research, these findings suggest that sleep disturbance may contribute to the development and maintenance of CIPN.

## 4. Implications for CIPN Prevention and Management

**Figure 2:**
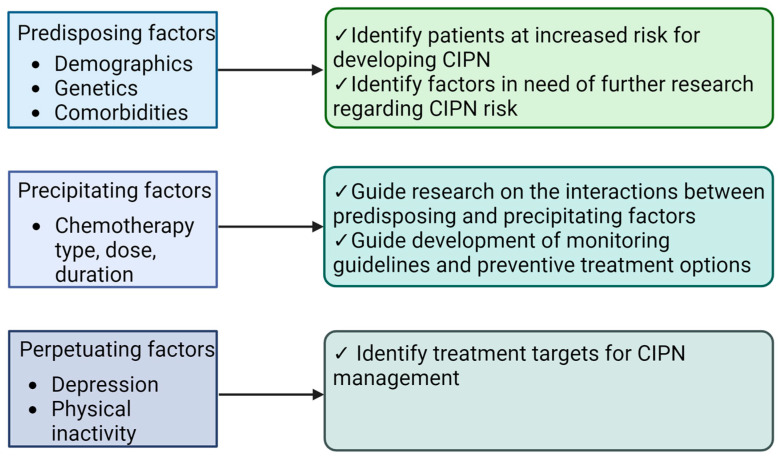
Potential applications of 3P CIPN model (Created using Biorender).

The proposed conceptual 3P framework comprises three domains (predisposing, precipitating, and perpetuating factors) that contribute to the development and maintenance of CIPN among cancer patients treated with chemotherapy. Within each domain, we have outlined specific theoretical and conceptually informed factors that may relate to CIPN (Figure 2). We anticipate several clinical applications of this framework that could improve CIPN management for cancer patients.

Predisposing factors outlined in this framework can be used to identify patients who have a higher risk for developing CIPN, particularly by age and clinical characteristics (e.g., pre-existing peripheral neuropathy and overall symptom burden); further research is needed regarding genetic predispositions, gender, and racial/ethnic groups. Similarly, precipitating factors related to particular chemotherapy regimens may carry unique risks for CIPN development and trajectories. Known precipitating factors include the type of chemotherapy agent, dosage, and duration of treatment. Currently, there are no comprehensive risk assessment tools for identifying patients at high risk for CIPN, and there are no preventative steps that mitigate this risk. However, the development of such a tool would enhance the identification of patients who would benefit from focused education and informed decision-making about their treatment course. Additionally, a further understanding of the interaction of predisposing and precipitating factors would help to enhance personalized medicine, allowing medical decision-making and recommended interventions to be more effectively tailored to the unique needs of individuals based on their predicted response to chemotherapy.

The major precipitating factor of CIPN is the delivery of chemotherapy to peripheral nerves, which may be mitigated via cryotherapy or compression therapy during chemotherapy. Cryotherapy is proposed to prevent CIPN via vasoconstriction induced by hypothermia, which results in lower exposure of peripheral nerves to causative chemotherapeutic agents. However, the few studies investigating the efficacy of cryotherapy in CIPN prevention or treatment are small, retrospective, or uncontrolled [99,106]. Clinical application of cryotherapy may also be limited because it is uncomfortable for patients; in a study of 180 patients receiving chemotherapy, 34% discontinued cryotherapy because of discomfort [107].

Compression therapy is proposed to function similarly to cryotherapy. Physical compression of peripheral blood vessels results in limited blood flow and, therefore, limited chemotherapy delivery to distal peripheral nerves. However, preliminary data regarding the efficacy of compression therapy are mixed. One study by Tsuyuki et al. [108] demonstrated improvement in sensory and motor neuropathy related to CIPN, whereas a subsequent study showed no improvement in rates of CIPN development [109]. Because of the limited evidence base, no agents or interventions are currently recommended to prevent CIPN [99]. More research is needed to identify patients at risk for the development of CIPN and to develop effective strategies for the prevention and management of CIPN.

Perpetuating factors can offer insight into psychological, cognitive, and behavioral modifications that could be treatment targets for CIPN management. Therefore, it is necessary to consider comprehensive pain management approaches that include non-pharmaceutical strategies intended to complement patients’ pharmacological regimens. Currently, ASCO guidelines only recommend duloxetine as a pharmacologic option for CIPN management, but there are a few additional evidence-based options to offer patients [1]. Exercise during chemotherapy is a promising treatment option with limited toxicity; in preliminary studies, it reduced CIPN-related symptoms of hot/cold sensations in the hand and feet but did not reduce numbness or tingling [99,110]. Preliminary data for acupuncture for relieving symptoms of CIPN are also promising, with studies demonstrating sustained improvement in pain and quality of life 14 weeks after an acupuncture intervention [111] and improvements in patient-reported neurotoxicity and pain [99,112].

Cutaneous electroanalgesia is another promising treatment for CIPN [99]. Transcutaneous electrical nerve stimulation (TENS) is a form of cutaneous electroanalgesia that inhibits the transmission of nociceptive signals from nerve fibers, thus decreasing pain perception. TENS has also been demonstrated to facilitate endogenous opioid release [113]. Cochrane systematic reviews of TENS for chronic pain showed mixed results of efficacy; additionally, many studies were subject to significant bias, which limits our ability to draw strong conclusions regarding the efficacy of TENS [113]. Another form of cutaneous electroanalgesia is scrambler therapy, which delivers electrical stimulation to the skin and produces non-noxious sensations instead of painful ones, thus reducing central sensitization to pain; however, its specific mechanism of action has yet to be fully elucidated [113]. Scrambler therapy has been demonstrated to reduce chronic pain [114], neuropathic pain [115,116], and reduced the use of analgesic drugs by up to 75% [113,114,115,116].

Though these modalities may relieve the symptom burden of CIPN, further study is needed to better understand their efficacy and potential toxicities [99]. Sleep disturbance, fatigue, and low physical activity, all of which were identified via this 3P framework, along with the distress associated with CIPN, are additional potential treatment targets for future intervention development. One such approach that may comprehensively target each of these factors is cognitive-behavioral therapy (CBT), which highlights the relationships among emotions, thoughts, and behaviors. Formal therapist-led CBT-based programs have been developed to treat chronic pain, depression, anxiety, insomnia, and cancer-related fatigue. Components of CBT interventions typically include psychoeducation, graded exercise and behavioral activation, engagement in healthy behaviors (e.g., sleep hygiene), relaxation training, cognitive restructuring, and other treatment targets. Though much of the CBT literature on pain management is focused on non-cancer patients, similar interventions may be of use for cancer patients with CIPN. There has been a single investigation into a self-guided online CBT intervention for cancer patients with chronic, painful CIPN, which demonstrated improvement in “worst” pain scores compared to usual care [117] However, it will be important for future CBT-based CIPN interventions to address cancer-specific factors related to diagnosis, treatment, and survivorship (e.g., fear of recurrence). Future research in this area may lead to targeted treatments that enhance effective CIPN management for cancer patients.

## 5. Conclusions

The proposed 3P model provides a conceptual framework to advance our understanding of the predisposing, precipitating, and perpetuating factors that influence CIPN. This framework can guide the development of effective interventions for CIPN by suggesting modifiable psychological and behavioral treatment targets that may mitigate the impact of CIPN for cancer patients. However, although the literature outlined in this framework is extensive, this model is not exhaustive. Given that CIPN is currently poorly understood, there are likely several contributing factors in each domain that influence the development and trajectory of CIPN symptoms. Consequently, future investigations may lead to further refinements of the framework and the likely identification of additional factors. Additionally, though we discuss each factor independently, it is likely that multiple factors across domains interact to maximize the risk of CIPN development for particular patient groups. Future research should strive to evaluate the interactions among these factors and provide a comprehensive risk assessment tool for patients planning to undergo chemotherapy.
